# Draft genome sequences of *Pseudomonas chengduensis* strain BW1 and *Sphingobium* sp. strain MK2 isolated from oil sands process-affected water

**DOI:** 10.1128/mra.00677-24

**Published:** 2024-12-19

**Authors:** Philemon Chinemezu Anuforo, Birgit Würz, Lukas Y. Wick, René Kallies

**Affiliations:** 1 Department of Applied Microbial Ecology, Helmholtz Centre for Environmental Research UFZ, Leipzig, Germany; 2 German Environment Agency, Section Microbial Risks, Berlin, Germany; Indiana University Bloomington, Bloomington, Indiana, USA

**Keywords:** *Pseudomonas*, *Sphingobium*, draft genome, oil sands process-affected water, phenanthrene degradation

## Abstract

Draft genomes of two phenanthrene-degrading bacterial isolates from oil sands process-affected water (OSPW) in Alberta, Canada were sequenced. Both isolates grew in close association on agar plates and were difficult to obtain axenically. They represent novel *Pseudomonas chengduensis* and *Sphingobium* sp. strains with genomes of 5.5 and 4.1 Mbases length, respectively.

## ANNOUNCEMENT

Oil sands process-affected water (OSPW) contains complex hydrocarbon mixtures, including the polycyclic aromatic hydrocarbon (PAH), phenanthrene (1). We here report the isolation and characterization of two OSPW bacteria (2) from the Fort McMurray region (Alberta, CA), utilizing phenanthrene as their sole carbon and energy source. Applying sterile handling, they were enriched by shaking solid phenanthrene (50 mg, >98%, Merck, Germany) in 50 mL OSPW for 9 months using culture flasks with vented caps. Evaporation losses were counteracted by minimal medium (MM [3]) addition. Aliquots were serially diluted in 10 mM phosphate-buffered saline (PBS) and dilutions cultivated on MM agar with 20 mg solid phenanthrene. Single colonies were separated on 1:10 diluted Luria–Bertani (1:10 LB) agar and colonies successively passaged on fresh phenanthrene MM agar (3). Purification obtained gray-white (strain BW1) and yellow (strain MK2) colonies consistently growing in close association. Axenic strains were obtained after serial dilution in 10 mM PBS and plating on 1:10 LB agar. Both strains grew independently on phenanthrene MM agar or liquid MM with phenanthrene (1 g L^−1^).

DNA was extracted from 1:10 LB grown cells using a NucleoSpin Microbial DNA Mini Kit (Machery and Nagel, Dueren, Germany). Sequencing libraries were prepared using a NEBNext Ultra II FS DNA Library Prep Kit (New England Biolabs, Ipswich, MA). Illumina sequencing was performed on a NextSeq platform (StarSEQ GmbH, Mainz, Germany) using NextSeq 500/550 v2.5 chemistry with 150 cycles, resulting in 2 × 5,603,469 (strain BW1) and 6,439,169 (strain MK2) 150 bp paired-end reads. Raw Illumina reads were trimmed using Trimmomatic version 0.39 and filtered for adapter sequences and low-quality bases using a sliding window approach (4 bp window, trimming if average quality dropped below 20). Sequences < 36 bp were discarded (4). High-quality reads were assembled *de novo* using SPAdes (version 3.15.5) with default parameters and options to minimize mismatches and short indels (5, 6). Separate genome assembly workflows were employed for strains BW1 and MK2 to avoid cross-contamination. The resulting scaffolds were submitted to the National Center for Biotechnology Information (NCBI) Prokaryotic Genome Annotation Pipeline (PGAP, version 6.6), where the coding sequences, tRNAs, rRNAs, and other features were identified by the GeneMarkS-2 + method and reference protein sets (7–9). Each assembly underwent an automatic contamination screen by NCBI during the GenBank submission process using the Foreign Contamination Screen tool. Contaminants were either trimmed or excluded automatically by the NCBI pipeline. Manual intervention was performed to remove remaining contaminants from the assembly, ensuring high-quality final genome sequences (10) (Table 1). Taxonomic classification was based on a 16S rRNA gene analysis and the NCBI prokaryotic genome annotation pipeline (11, 12). The latter identified strain BW1 as 96.769% identical by average nucleotide identity to the type genome of *Pseudomonas chengduensis*, with 81.5% coverage of the genome. *Sphingobium* sp. MK2 was identified similarly using the 16S rRNA gene sequence analysis and average nucleotide identity calculations, confirming its taxonomic placement within the *Sphingobium* genus. The *Pseudomonas* and *Sphingobium* species are known PAH degraders (13). Although their synergistic co-existence in OSPW remains undescribed, bacterial co-cultures in OSPW likely perform better than pure cultures (14).

**TABLE 1:** Sequencing data and genome characteristics for *Pseudomonas chengduensis* BW1 and *Sphingobium* sp. MK2

Characteristics	Findings	
	*Pseudomonas chengduensis* BW1	*Sphingobium* sp. MK2
Read length (bases)	2 × 150	2 × 150
Number of read pairs	5,603,469	6,439,169
Number of filtered read pairs	5,388,333	6,202,392
Total genome length (bp)	5,476,076	4,140,971
GC content (%)	62.5	62.3
Average coverage (fold)	117	123
Number of contigs	204	118
N50 (bp)	383,039	88,159
Max contig length	638,087	306,336
Min contig length	201	508
L50	6	15
Number of CDS	5,182	4,041
Number of CDS with predicted function	5,111	3,968
Number of tRNAs	56	67
SRA accession number	SRR29064398	SRR29064397
BioSample number	SAMN39250567	SAMN39584109
GenBank accession number	JAYXUA000000000	JAZDXA000000000

## Data Availability

Raw sequencing reads were deposited at the NCBI Sequence Read Archive (SRA). Sequencing reads and the assembled genome data are available under BioProject PRJNA1060937 with BioSample SAMN39250567 and accession no. SRR29064398 (*Pseudomonas chengduensis* BW1) and BioSample SAMN39584109 and accession no. SRR29064397 (*Sphingobium* sp. MK2). The whole genome shotgun sequencing projects are available at GenBank under accession nos. JAYXUA000000000 (*Pseudomonas chengduensis* BW1) and JAZDXA000000000 (*Sphingobium* sp. MK2). Both strains are deposited in the microbial strain collection of the Helmholtz Centre for Environmental Research UFZ, Leipzig, Germany.
